# Host-Specific Metabolic Engineering for Microbial Production of Fucosyllactoses

**DOI:** 10.4014/jmb.2608.08032

**Published:** 2026-10-15

**Authors:** Bo Min Kim, You Jin Jang, Hee Yeon Song, Suk-Chae Jung, Jonghyeok Shin, Jae Won Lee

**Affiliations:** 1 Department of Biotechnology, Sungshin Women’s University, Seoul, Republic of Korea; 2 School of Biopharmaceutical and Medical Sciences, Sungshin Women’s University, Seoul, Republic of Korea; 3 Department of Chemical and Biomolecular Engineering, University of Illinois Urbana-Champaign, Urbana, IL, USA; 4 Department of Food Science and Biotechnology, College of Life Sciences, Sejong University, Seoul, Republic of Korea; 5 Carbohydrate Bioproduct Research Center, Sejong University, Seoul, Republic of Korea

**Keywords:** Human milk oligosaccharides (HMOs), 2′-fucosyllactose (2′-FL), 3-fucosyllactose (3-FL), Metabolic engineering, Microbial cell factory

## Abstract

Human milk oligosaccharides (HMOs) are a diverse group of bioactive glycans that play important roles in infant health by modulating the gut microbiota, inhibiting pathogen adhesion, and promoting immune development. Among them, 2′-fucosyllactose (2′-FL) and 3-fucosyllactose (3-FL) have attracted considerable attention owing to their established health benefits and increasing applications in infant formula and functional foods. Microbial biosynthesis has emerged as a sustainable and scalable alternative to chemical synthesis and enzymatic production. This review summarizes recent advances in microbial FL production using *Escherichia coli*, *Saccharomyces cerevisiae*, and *Yarrowia lipolytica*, with emphasis on host-specific metabolic engineering strategies. Key engineering approaches, including GDP-L-fucose biosynthesis, lactose uptake, fucosyltransferase (FT) optimization, transporter engineering, and fermentation process development, are systematically discussed and compared across microbial hosts. The review highlights the distinct advantages and limitations of bacterial and yeast platforms, including product secretion, intracellular FL accumulation, carbon flux distribution, and redox balance. Finally, current bottlenecks and future engineering opportunities are discussed, including transporter optimization, host-specific metabolic regulation, and industrial process development. These advances provide valuable insights into the development of efficient, safe, and economically viable microbial cell factories for the sustainable production of FL and other fucosylated HMOs.

## Introduction

Human milk oligosaccharides (HMOs) are abundant components of human milk and play key roles in gut health, immune modulation, and protecting against pathogen colonization, particularly during infancy [1]. Among them, fucosylated HMOs have attracted particular attention due to their distinct biological activities, including antiviral, anti-inflammatory, and immunomodulatory effects, which differ from those of common monosaccharides and non-fucosylated oligosaccharides [2]. The outstanding physiological functions of fucosylated HMOs have attracted considerable interest not only in academic research but also in industrial applications. Considerable efforts have been made to incorporate these compounds into infant formula to better mimic the nutritional profile of human milk [3]. In addition, co-administration of HMOs with probiotics is being explored to achieve synergistic effects on gut health and immune modulation [4].

Beyond infant nutrition, fucosyllactoses (FLs), particularly 2′-fucosyllactose (2′-FL), are being explored for potential applications in functional foods, prebiotic formulations, and other health-oriented nutritional products [5]. Clinical studies have demonstrated that 2??FL, alone or in combination with lacto-N-neotetraose (LNnT), is well tolerated and can modulate the intestinal microbiota in adults, supporting its potential applications beyond infant formula [6, 7]. In contrast, clinical evidence supporting the applications of 3-fucosyllactose (3-FL) beyond infant nutrition remains limited [2].

The growing interest in FL applications beyond infant nutrition highlights the need for scalable and cost-effective production platforms. However, conventional approaches face several practical limitations. Direct extraction from human milk is constrained by supply, low yields, and high cost [8]. Chemical synthesis offers structural precision but involves complex multi-step processes and expensive purification [9]. Enzymatic synthesis requires mild reaction conditions but is constrained by enzyme costs, substrate specificity, and challenges in process optimization [10]. To address these limitations, microbial biosynthesis has emerged as a scalable and efficient alternative, enabling selective production through pathway reconstruction in genetically tractable hosts [11, 12].

To date, microbial production of fucosylated HMOs has focused on bacterial systems such as *Bacillus subtilis*, *Corynebacterium glutamicum*, and *Escherichia coli* [13]. These hosts are widely used due to their metabolic versatility, genetic tractability, and ability to utilize diverse carbon sources [12, 14, 15]. However, *B. subtilis* and *C. glutamicum* require extensive reconstruction of the GDP-L-fucose biosynthetic pathway and additional host-specific optimization to achieve efficient FL production [12, 16]. In contrast, *E. coli* has become the leading bacterial platform for FL production owing to its well-characterized metabolism, extensive engineering toolbox, and demonstrated scalable FL production [11, 14, 17, 18].

Substantial efforts have also focused on yeast-based production systems. Several yeast species, *Saccharomyces cerevisiae*, *Komagataella phaffii* (formerly Pichia pastoris), and *Yarrowia lipolytica*, have been explored for fucosylated HMO production [19]. Among these, *S. cerevisiae* and *Y. lipolytica* have emerged as major yeast platforms with distinct advantages. *S. cerevisiae* benefits from well-established genetic tools, extensive industrial use, and generally recognized as safe (GRAS) status, supporting its application in scalable FL production [20-24]. In contrast, *Y. lipolytica* combines robust carbon metabolism, broad substrate utilization, and high-cell-density cultivation. Recent studies have reported efficient production of 2′-FL in engineered *Y. lipolytica*, highlighting its potential as a scalable yeast platform [25-27]. Although *K. phaffii* has also been investigated for 2′-FL production owing to its strong heterologous expression capacity, its application to fucosylated HMO production remains comparatively limited [28-30].

Despite the structural diversity of fucosylated HMOs, only a subset has been extensively explored for microbial production [31]. Among these, 2′-FL and 3-FL are the most abundant and well-known fucosylated HMOs, with established physiological functions and commercial relevance [17]. Their relatively simple structures and well-defined biosynthetic pathways make them ideal model compounds for developing and evaluating microbial production strategies. Accordingly, these molecules serve as representative targets for elucidating key metabolic engineering principles and guiding industrial-scale applications.

This review summarizes recent advances in the microbial production of fucosylated HMOs and discusses future directions for their industrial development. We focus on *E. coli*, *S. cerevisiae*, and *Y. lipolytica* as major bacterial and yeast platforms. Using 2′-FL and 3-FL as representative products, we comparatively examine host-specific metabolic constraints and engineering strategies, with particular emphasis on pathway engineering, strain optimization, and fermentation process development. Finally, we highlight current limitations and future opportunities for scalable FL production.

## Regioisomer-Specific Biological Functions of 2′-FL and 3-FL

Although 2′-FL and 3-FL are both trisaccharides composed of lactose and a single fucose residue, their biological activities can differ according to the position of fucosylation. In 2′-FL, fucose is linked to the C2 position of the galactose residue through an α-1,2 linkage, whereas in 3-FL, fucose is attached to the C3 position of the glucose moiety through an α-1,3 linkage. This positional difference alters the spatial presentation of the fucosylated glycan epitope and may influence host–glycan interactions in the intestinal environment [32, 33] (Fig. 1A).

Comparative studies suggest that these structural differences are associated with distinct effects on intestinal barrier regulation and immune responses. In IL-6-induced Caco-2 epithelial barrier dysfunction and DSS-induced colitis models, both 2′-FL and 3-FL improved epithelial barrier integrity, as shown by restored transepithelial electrical resistance, reduced FITC-dextran permeability, and attenuation of colitis-associated phenotypes [34]. However, 2′-FL showed stronger effects on tight-junction regulation than 3-FL, including greater restoration of ZO-1 and occludin and stronger suppression of claudin-2 [34-37]. These findings suggest that the α-1,2-fucosylated structure of 2′-FL may confer stronger barrier-protective activity under inflammatory conditions (Fig. 1B).

Regioisomer-specific effects have also been reported in allergic inflammation models. In an ovalbumin-induced mouse model of hen’s egg allergy, dietary 3-FL reduced the T helper 2 (Th2) cell populations in mesenteric lymph nodes while increasing regulatory T cell (T_reg_) frequencies, suggesting modulation of the Th2/Treg balance. In contrast, 2′-FL reduced serum mouse mast cell protease-1, a marker of mucosal mast-cell activation, and increased cecal short-chain fatty acid (SCFA) concentrations, indicating a possible association with microbiota-derived metabolite responses [38-40] (Fig. 1C). In a house dust mite-induced allergic asthma model, both regioisomers reduced allergen-induced thymic stromal lymphopoietin (TSLP) and IL-8 release from bronchial epithelial cell–dendritic cell cocultures and decreased serum house dust mite-specific IgE. However, 3-FL showed broader effects on cytokine regulation, including the preservation of TGF-β secretion and reductions in IL-4, IL-13, and pulmonary IFN-γ levels [41].

Together, these studies indicate that 2′-FL and 3-FL can exert both overlapping and distinct biological activities. Whereas 3-FL showed more pronounced effects on T-cell polarization and cytokine regulation, 2′-FL more strongly influenced mast-cell degranulation and SCFA-associated responses in the food allergy model. These findings suggest that fucosyl linkage position may shape immune regulation in a tissue- and model-dependent manner, underscoring the need for microbial production strategies to consider not only FL yield but also regioisomer specificity when developing FLs for targeted health applications.

## Metabolic Engineering Strategies for FL Production

### Biosynthetic pathways for FL production in *E. coli*, *S. cerevisiae*, and *Y. lipolytica*

The biosynthesis of fucosylated HMOs, such as 2′-FL and 3-FL, requires intracellular GDP-L-fucose as the activated fucose donor, lactose as the acceptor substrate, and a fucosyltransferase (FT) that catalyzes the transfer of fucose from GDP-L-fucose to lactose. The availability and functionality of these components vary substantially among microbial hosts and therefore determine host-specific strategies for pathway construction and optimization (Fig. 2).

GDP-L-fucose availability differs substantially among microbial hosts. *E. coli* is one of the most widely used platforms for FL production because it possesses an endogenous *de novo* pathway for GDP-L-fucose biosynthesis. In this pathway, central carbon metabolites are converted to GDP-D-mannose through the sequential actions of mannose-6-phosphate isomerase (ManA), phosphomannomutase (ManB), and mannose-1-phosphate guanylyltransferase (ManC). GDP-D-mannose is subsequently converted to GDP-L-fucose by GDP-D-mannose-4,6-dehydratase (Gmd) and GDP-L-fucose synthase (WcaG). The resulting GDP-L-fucose serves as the activated fucose donor for FL biosynthesis [17] (Fig. 2A).

In contrast to *E. coli*, *S. cerevisiae*, and *Y. lipolytica* do not naturally possess a complete GDP-L-fucose biosynthetic pathway. However, both yeasts contain an endogenous GDP-D-mannose pathway that supports cell wall biosynthesis and protein glycosylation. In this pathway, fructose-6-phosphate is sequentially converted to mannose-6-phosphate, mannose-1-phosphate, and GDP-D-mannose by mannose-6-phosphate isomerase, phosphomannomutase, and mannose-1-phosphate guanylyltransferase, respectively [42]. Therefore, FL production in both yeasts requires reconstruction of the downstream GDP-L-fucose biosynthetic module by introducing heterologous Gmd and WcaG, or functionally equivalent enzymes [22, 43] (Fig. 2B).

Intracellular lactose availability is another important determinant of FL production because lactose serves as the acceptor substrate for fucosylation. *E. coli* possesses the native lactose permease LacY, which transports lactose into the cell, and β-galactosidase LacZ, which hydrolyzes lactose into glucose and galactose. Accordingly, FL-producing *E. coli* strains generally retain or enhance *lacY* expression to support lactose uptake while deleting or inactivating *lacZ* to prevent degradation of the acceptor substrate [1, 17, 44-46].

In contrast, *S. cerevisiae* and *Y. lipolytica* lack efficient native lactose uptake and therefore require the introduction of a heterologous lactose transporter, most commonly Lac12 from *Kluyveromyces lactis* [21, 22, 25]. Because lactose must remain intact for fucosylation, heterologous β-galactosidase activity is generally unnecessary for FL production. Thus, strain engineering to improve lactose availability in *E. coli* primarily involves balancing lactose uptake and degradation, whereas in yeast, it requires establishing and optimizing heterologous lactose transport.

The final fucosylation step requires an FT with the appropriate linkage specificity. α-1,2-FTs transfer fucose to the galactose residue of lactose to produce 2′-FL, whereas α-1,3-FTs transfer fucose to the glucose residue of lactose to produce 3-FL [17]. Because *E. coli*, *S. cerevisiae*, and *Y. lipolytica* generally lack endogenous FT activity suitable for efficient FL production, heterologous expression of an appropriate FT is essential [1, 25, 47]. However, FT performance is strongly influenced by enzyme origin, catalytic activity, soluble expression, subcellular localization, gene dosage, and compatibility with the host [48-50]. Therefore, FT screening and engineering are required in addition to optimizing GDP-L-fucose and lactose availability.

Thus, *E. coli* and the two yeast hosts provide different metabolic starting points for FL production with respect to GDP-L-fucose biosynthesis, lactose uptake, and FT expression. *E. coli* possesses an endogenous GDP-L-fucose pathway and a native lactose transport system, although competing GDP-L-fucose consumption and lactose degradation must be suppressed. In contrast, *S. cerevisiae* and *Y. lipolytica* require reconstruction of the GDP-L-fucose biosynthetic module as well as the introduction of a heterologous lactose transporter. In all three hosts, the expression and optimization of α-1,2- or α-1,3-FTs are required depending on the target FL isomer. These host-specific differences provide the basis for distinct engineering strategies to improve FL production.

### Metabolic Engineering of *E. coli* for FL Production

**GDP-L-fucose supply and pathway optimization in *E. coli*.** Increasing the intracellular availability of GDP-L-fucose is a primary objective in the construction of FL-producing *E. coli*. GDP-L-fucose can be produced through either the endogenous *de novo* pathway or a heterologous salvage pathway (Fig. 2A). The *de novo* pathway produces GDP-L-fucose from central carbon metabolites without requiring external L-fucose but depends on sufficient precursor flux, GTP availability, and NADPH supply. In contrast, the salvage pathway converts exogenous L-fucose to GDP-L-fucose through bifunctional L-fucose kinase/L-fucose-1-phosphate guanylyltransferase (Fkp), commonly derived from *Bacteroides fragilis* [32, 51-53]. Although the salvage pathway provides a relatively direct route to GDP-L-fucose, its dependence on externally supplied L-fucose may limit its economic feasibility for large-scale production.

Overexpression strategies have been widely used to reinforce GDP-L-fucose biosynthesis. In the *de novo* pathway, *manB*, *manC*, *gmd*, and *wcaG* are commonly overexpressed to increase the conversion of central carbon metabolites to GDP-L-fucose [45, 54-56]. Because the final WcaG-catalyzed reduction requires NADPH, cofactor engineering has been used to reinforce the intracellular reducing power supply. Lee *et al*. compared the overexpression of *zwf*, *icd*, and *maeB*, encoding glucose-6-phosphate dehydrogenase, isocitrate dehydrogenase, and NADP-dependent malic enzyme, respectively, in a GDP-L-fucose-producing *E. coli* strain [57]. In batch cultivation, *zwf* and *icd* overexpression increased GDP-L-fucose accumulation by 46% and approximately 31%, whereas *maeB* overexpression reduced it by 24%. Among the genes tested, *zwf* overexpression was the most effective, yielding 235.2 mg/L GDP-L-fucose under optimized pH-stat fed-batch conditions, a 21% increase over the control.

More recently, M. Li *et al*. further evaluated transhydrogenase-based redox engineering for 2′-FL production. PntAB, a membrane-bound proton-translocating pyridine nucleotide transhydrogenase, promotes NADPH formation by transferring reducing equivalents from NADH to NADP^+^, whereas UdhA is a soluble pyridine nucleotide transhydrogenase involved in balancing the intracellular NAD(H) and NADP(H) pools [44]. Individual overexpression of *zwf* and *pntAB* increased 2′-FL production to 1.71 and 1.62 g/L, corresponding to improvements of 17.9% and 11.7%, respectively. Co-expression of *pntAB* and *udhA* further increased the titer to 2.06 g/L, 42.1% above that of the control strain, indicating that coordinated transhydrogenase expression can improve intracellular redox balance for 2′-FL biosynthesis. In addition, guanine nucleotide metabolism has been enhanced by overexpressing *gsk*, *gmk*, and *ndk*, encoding guanosine kinase, guanylate kinase, and nucleoside diphosphate kinase, respectively. These enzymes support guanine nucleotide interconversion from guanosine through GMP and GDP to GTP, thereby increasing the nucleotide supply required for GDP-L-fucose biosynthesis. Consistent with this strategy, Ni *et al*. overexpressed *zwf* and *pntAB* to enhance NADPH supply and *gsk* to increase GTP availability in an engineered *E. coli* strain expressing a modified α-1,3-FT, resulting in 35.72 g/L 3-FL in a 5-L bioreactor with a yield of 0.82 mol/mol lactose [58].

In the salvage pathway, heterologous expression of *fkp* enables the conversion of exogenous L-fucose to GDP-L-fucose. However, efficient production also depends on the uptake of extracellular L-fucose. In *E. coli*, L-fucose is transported across the cytoplasmic membrane by FucP, an L-fucose/H^+^ symporter [59]. Baumgärtner *et al*. demonstrated salvage pathway-based 2′-FL production in *E. coli* by deletion of *fucI* and *fucK* to prevent L-fucose degradation while retaining the native FucP transport system for L-fucose uptake [60]. Furthermore, overexpression of *fucP* increased lactodifucotetraose production to 0.9 g/L, representing a 6.9-fold increase in an engineered *E. coli* strain [61]. These findings highlight the importance of coordinating L-fucose uptake with its intracellular conversion and the suppression of competing catabolic pathways for efficient salvage pathway-based fucosylated HMO production.

Zhai *et al*. introduced *fkp* from *B. fragilis* into *E. coli* and strengthened GTP supply by overexpressing *gpt* together with the previously described *gmk* and *ndk*. Gpt, a guanine phosphoribosyltransferase, provides an alternative entry into guanine nucleotide biosynthesis by converting guanine and phosphoribosyl pyrophosphate to GMP, which is subsequently converted to GDP and GTP by Gmk and Ndk [62]. This strategy increased GDP-L-fucose accumulation to 4.6 ± 0.22 μmol/g DCW in shake-flask cultivation and 6.6 ± 0.14 μmol/g DCW during fed-batch cultivation.

Gene deletion has also been used to block competing pathways and preserve GDP-L-fucose. Deletion of *wcaJ*, which encodes UDP-glucose:undecaprenyl-phosphate glucose-1-phosphate transferase, interrupts colanic acid (CA) biosynthesis and prevents GDP-L-fucose consumption through this pathway [63]. Regulatory and cell envelope-related gene deletions have also been combined with *wcaJ* deletion to further increase intracellular GDP-L-fucose accumulation [64]. For example, deletion of *lon*, which encodes the Lon protease, stabilizes the transcriptional activator RcsA by preventing its degradation, thereby activating CA biosynthesis [65]. Similarly, deletion of *waaF*, encoding ADP-heptose heptosyltransferase II, activates the Rcs phosphorelay system and induces CA biosynthesis. When combined with *wcaJ* deletion, these perturbations activate upstream flux while blocking downstream consumption, thereby redirecting metabolism toward GDP-L-fucose accumulation. The resulting Δ*waaF*Δ*wcaJ* strain accumulated 1.39 mg GDP-L-fucose/g cell without additional overexpression of the *de novo* pathway genes, exceeding the 1.02 mg/g cell obtained in the corresponding overexpression strain [66].

Further improvement has been achieved by targeting competing reactions at multiple precursor levels. Mengli Li *et al*. evaluated reactions associated with fructose-6-phosphate, GDP-D-mannose, and GDP-L-fucose [14]. In this context, *pfkA* directs fructose-6-phosphate into glycolysis, whereas *nudD* diverts GDP-D-mannose away from GDP-L-fucose biosynthesis. Combined deletion of *wcaJ*, *nudD*, *pfkA*, and *lon* redirected metabolic flux toward the GDP-L-fucose pathway, resulting in 6.24 g/L 2′-FL and 3.56 g/L 3-FL in shake-flask cultivation. These findings indicate that simultaneous control of precursor branching, nucleotide-sugar degradation, and downstream consumption can support the production of both FL isomers. In the salvage pathway, deletion of L-fucose catabolic genes preserves externally supplied L-fucose for Fkp-mediated GDP-L-fucose synthesis. Deletion of *fucI* and *fucK*, encoding L-fucose isomerase and L-fuculokinase, respectively, blocks the native L-fucose degradation pathway. Additional removal of *araA* and *rhaA*, which encode promiscuous sugar isomerases with activity toward L-fucose, further reduces competing L-fucose conversion. Chin *et al*. constructed an engineered *E. coli* BL21star(DE3) strain expressing Fkp from *B. fragilis* with deletions of *lacZ*, *fucI*, and *fucK* [51]. The resulting strain produced 23.1 g/L extracellular 2′-FL in fed-batch fermentation. Building on this strain, Jung *et al*. additionally deleted *araA* and *rhaA*, increasing the 2′-FL titers approximately twofold to 47.0 g/L and the molar conversion yield by 44%, from 0.36 to 0.52 mol 2′-FL/mol L-fucose [52]. A related strategy was applied to 3-FL production by Choi *et al*., who used an *E. coli* BL21(DE3) strain deficient in *fucI* and *fucK* and co-expressed *fkp*, *gsk*, and an engineered α-1,3-FT [32]. These modifications enabled the production of 4.6 g/L 3-FL, with a productivity of 0.076 g/L/h and a specific yield of 0.5 g/g DCW.

Integrated strategies that combine pathway reinforcement, competing-pathway deletion, and cofactor engineering have yielded further improvements. Wan *et al*. divided the *de novo* GDP-L-fucose pathway into an upstream ManB–ManC module and a downstream Gmd–WcaG module, and balanced their expression through plasmid copy number and ribosome-binding site (RBS) optimization [67]. Low expression of the upstream module, combined with high expression of the downstream module, promoted efficient conversion of GDP-D-mannose to GDP-L-fucose. Further deletion of *wcaJ* prevented GDP-L-fucose consumption via CA biosynthesis, whereas overexpression of *zwf* and *gsk* enhanced regeneration of NADPH and GTP, respectively. These modifications increased intracellular GDP-L-fucose accumulation to 18.33 mg/L in shake-flask cultivation, and the final strain achieved 106.21 mg/L, corresponding to 4.28 mg/g DCW, during fed-batch cultivation. Although this strain was designed for GDP-L-fucose production rather than FL biosynthesis, subsequent studies applied related precursor-engineering principles to improve 2′-FL production. Li *et al*. reconstructed and transcriptionally balanced both the *de novo* and salvage pathways, introduced *fkp*, and inactivated bypass genes including *fucI*, *fucK*, and *wcaJ* [56]. The final strain produced 2.62 g/L 2′-FL in shake-flask cultivation and 14.1 g/L in fed-batch cultivation. Liu *et al*. similarly strengthened the *de novo* pathway by replacing the native promoters of the *manC*–*manB* and *gmd*–*wcaG* clusters with the strong constitutive promoter (P_J23119_) and increasing the dosage of chromosomal α-1,2-FT (*wbgL* from *E. coli* O126:H27) [45]. These modifications increased the 2′-FL titers from 3.92 to 9.06 g/L in shake-flask cultivation.

Collectively, these studies indicate that efficient GDP-L-fucose supply requires coordinated reinforcement of precursor synthesis, nucleotide and redox cofactor availability, and suppression of competing reactions. The optimal strategy differs between the *de novo* and salvage pathways. The *de novo* pathway depends primarily on central carbon flux, NADPH supply, and prevention of GDP-L-fucose consumption, whereas the salvage pathway requires efficient L-fucose conversion and elimination of competing L-fucose catabolism.

**Lactose availability and acceptor-substrate engineering in *E. coli*.** In addition to the GDP-L-fucose supply, sufficient intracellular lactose is required because lactose serves as the acceptor-substrate for the FT-catalyzed reaction. Native *E. coli* imports lactose via the lactose/proton symporter LacY but hydrolyzes it to glucose and galactose via the β-galactosidase LacZ. Consequently, strain engineering generally aims to retain or enhance LacY-mediated uptake while reducing LacZ-dependent degradation, thereby preserving intracellular lactose for fucosylation.

An early strategy involved attenuating LacZ activity without disrupting LacY-dependent lactose transport. Chin *et al*. replaced the endogenous *lac* operon of *E. coli* BL21star(DE3) with a modified operon containing *lacZΔM15* [68]. This modification decreased β-galactosidase activity while retaining lactose transport, increasing the 2′-FL yield approximately threefold. Combined with improved soluble expression of FucT2, the engineered strain produced 6.4 g/L 2′-FL in fed-batch fermentation, with a yield of 0.225 g 2′-FL/g lactose. A related strategy was used by Choi *et al*. [32] for salvage pathway-based 3-FL production. The *lacZΔM15* strain, combined with enhanced GTP supply and an engineered α-1,3-FT, produced 4.6 g/L 3-FL with a productivity of 0.076 g/L/h.

Complete *lacZ* deletion has subsequently been combined with disruption of GDP-L-fucose-consuming pathways in several *de novo* 2′-FL-producing strains [1, 44, 45]. M. Li *et al*. used a 2′-FL-producing strain as the control and showed that combined deletion of *lacZ* and *wcaJ* increased the 2′-FL titers from 0.22 to 1.45 g/L and the lactose-based yield from approximately 0.07 to 0.61 mol/mol, corresponding to 6.59- and 8.8-fold improvements, respectively [44].

Lactose availability can also be improved by strengthening *lacY* expression or modifying its transcriptional regulation. Park *et al*. replaced the native glucose-repressible *lac* promoter with the *tac* promoter, maintaining lactose transport under glucose-rich conditions [46]. When combined with structure-guided engineering of FutC, the resulting strain produced 40 g/L 2′-FL in fed-batch fermentation, with a productivity of 0.55 g/L/h. Chen *et al*. similarly enhanced lactose uptake in *E. coli* MG1655 by replacing the native *lacY* promoter with the strong constitutive promoter (P_J23119_) [69]. Combined with *lacZ* and *wcaJ* deletion and reinforcement of GDP-L-fucose biosynthesis, this lactose-transport engineering contributed to final 2′-FL titers of 76.9 and 50.1 g/L in the optimized plasmid-based and plasmid-free strains, respectively, during fed-batch fermentation. More recently, N. Li *et al*. further improved lactose utilization by introducing multiple chromosomal copies of *lacY* into *E. coli* BL21(DE3) [18]. Combined with reinforcement of GDP-L-fucose biosynthesis and additional engineering of product export, central carbon metabolism, and FT performance, the final strain produced 141.27 g/L 2′-FL after 45 h of fed-batch fermentation.

However, increasing lactose uptake is not universally beneficial. In an engineered *E. coli* C41(DE3) derivative, *lacY* overexpression transiently increased intracellular lactose but decreased biomass and 2′-FL production, which the authors attributed to possible cellular stress caused by excessive lactose uptake [1]. This interpretation is consistent with previous studies showing that excessive LacY-mediated lactose/proton symport can inhibit *E. coli* growth and impair cellular energetics, potentially through cytoplasmic acidification and disruption of the proton motive force [70-72]. These findings suggest that LacY expression should be balanced with intracellular lactose conversion rather than maximized independently.

An alternative strategy is to eliminate the requirement for exogenous lactose by constructing an intracellular lactose biosynthetic pathway. Parschat *et al*. established *de novo* lactose synthesis from sucrose by coupling UDP-D-galactose formation with the generation of free glucose [11]. GalTpm1141, a β-1,4-galactosyltransferase, catalyzed lactose formation by transferring galactose from UDP-D-galactose to free glucose, while Glf, an energy-independent glucose facilitator from *Zymomonas mobilis*, increased intracellular glucose availability through reuptake of extracellular glucose. These modifications improved the sucrose-to-2′-FL conversion yield from 0.12 to 0.18 mol/mol, and the final strain produced 64 g/L 2′-FL after 84 h of fed-batch fermentation.

Overall, lactose availability can be improved by attenuating or eliminating LacZ-dependent degradation, strengthening or deregulating LacY-mediated uptake, or constructing a *de novo* lactose biosynthetic pathway. Partial LacZ attenuation can preserve the organization and transport function of the native *lac* operon, whereas complete *lacZ* deletion more effectively prevents lactose hydrolysis. Nevertheless, lactose uptake must be coordinated with FT activity and product export to avoid intracellular imbalance.

**Heterologous expression and engineering of FTs in *E. coli*.** Most *E. coli* strains lack endogenous fucosylation activity, making heterologous expression of FTs essential for FL production. FTs transfer fucose from GDP-L-fucose to lactose. Specifically, α-1,2-FT and α-1,3-FT catalyze the synthesis of 2′-FL and 3-FL, respectively, enabling the biosynthesis of diverse fucosylated HMOs [73]. However, FT expression in *E. coli* is often limited by poor solubility [46], inclusion body formation [74], and low catalytic activity [75], reducing overall productivity [76].

To address these challenges, strategies such as fusion tags [68, 74, 75], site-directed mutagenesis [46, 74, 76], codon optimization [76], and structure-based protein engineering [22, 46] have been widely applied. Representative strategies to improve the solubility of α-1,2-FT involve fusion with solubility-enhancing tags, including thioredoxin A (TrxA), GroE small protein (GroES), maltose-binding protein (MBP), N-utilization substance protein A (NusA), and poly-aspartate tags, at the N- or C-terminus. Fusion of TrxA to α-1,2-FT (FutC from *H. pylori*) increased 2′-FL production by 6.39% compared with the untagged strain [74]. Similarly, N-terminal fusion of triple aspartate tags to α-1,2-FT (FucT2 from *H. pylori*) improved solubility and expression, resulting in a 2′-FL titer of 6.4 g/L in fed-batch fermentation, a 2.5-fold increase compared with the strain expressing untagged FucT2 (2.6 g/L) [68]. In addition, C-terminal fusion of septum site-determining protein (MinD) to α-1,2-FT (WbgL from *E. coli* O126:H27) enhanced protein stability and catalytic activity, increasing 2′-FL production by 24% from 4.16 to 5.14 g/L in batch fermentation [75]. Despite these improvements, further optimization of FT solubility, expression, and catalytic activity remains necessary to achieve higher FL productivity.

Following fusion of the TrxA tag to FutC, site-directed mutagenesis at key residues further enhanced enzymatic activity. The F40S/Q237S double mutant produced 3.53 g/L 2′-FL, a 1.55-fold increase over the control strain (2.28 g/L) [74]. These results indicate that the catalytic efficiency of FutC remains a major bottleneck in 2′-FL production, underscoring the need for systematic sequence- and structure-based engineering. Accordingly, Park *et al*. developed a rational engineering strategy integrating multiple sequence alignment and protein–substrate docking to improve both solubility and activity [46]. A quadruple mutant (F40S, Q150H, C151R, and Q239S) achieved 6.8 g/L 2′-FL in batch fermentation, representing a 2.4-fold increase over the wild type (2.8 g/L). Together, these studies demonstrate that combining solubility-enhancing fusion strategies with sequence- and structure-guided mutagenesis can improve α-1,2-FT performance and enhance 2′-FL production in *E. coli*.

Beyond α-1,2-FT engineering for 2′-FL production, Mengli Li *et al*. further optimized the expression of both α-1,2-FT FutC and α-1,3-FT M32 from *H. pylori* for 2′-FL and 3-FL production, respectively [14]. Through combinatorial optimization of RBS strength, fusion peptides, and gene copy number, the authors improved the production of both FL isomers. Among the tested RBSs, BBa_B0034 increased 2′-FL and 3-FL production to 7.87 and 5.26 g/L, respectively. N-terminal fusion with the PelB signal peptide, derived from pectate lyase B and commonly used for periplasmic targeting in *E. coli*, further increased the titers to 8.57 and 5.78 g/L. Subsequent copy-number optimization showed that strains carrying two copies produced the highest titers, reaching 9.36 g/L for 2′-FL and 6.28 g/L for 3-FL in shake-flask cultivation. Further increasing the copy number decreased biomass, which the authors attributed to the effects of increased plasmid length on cellular growth and metabolic activity. These results demonstrate the importance of optimizing FT expression while minimizing its adverse effects on host growth.

Chen *et al*. improved 3-FL production by chromosomally integrating four copies of the α-1,3-FT (Fut3Bc from *Neobacillus cucumis*) into the arsenite transporter (*arsB*), lactate dehydrogenase (*ldhA*), pyruvate oxidase (*poxB*), and mannose phosphotransferase system transporter complex (*manXYZ*) loci of *E. coli* [77]. The resulting strain produced 2.47 g/L of 3-FL, representing an 8.19-fold increase over the single-copy strain (0.3 g/L). This result demonstrates that increasing chromosomal FT copy number can improve 3-FL production while avoiding plasmid-dependent expression. In parallel, Choi *et al*. improved the soluble expression and catalytic activity of the α-1,3-FT FutA from *H. pylori* through codon optimization, C-terminal truncation, and site-directed mutagenesis [76]. The Δ52 FutA variant, in which the membrane-anchoring region and most of the C-terminal heptad repeats were removed, achieved a soluble expression level of 150–200 mg/L. Subsequent engineering generated the A128N/H129E/Y132I/S46F quadruple mutant, which exhibited a 15.5-fold increase in specific activity, from 0.08 to 1.24 U/mg.

These findings indicate that improving soluble expression and catalytic activity is important for α-1,3-FT engineering. However, enzyme performance in whole-cell systems is also influenced by structural features that affect folding, oligomerization, and intracellular activity.

Yu *et al*. systematically examined the effects of the C-terminal membrane-anchoring region and heptad repeats, seven-residue sequence motifs that form coiled-coil structures and facilitate enzyme dimerization, in three *H. pylori*-derived α-1,3-FTs [22]. Removal of the membrane-anchoring region improved 3-FL production, whereas extensive truncation of the heptad repeats reduced enzyme performance. In contrast, elongation of the heptad repeats after removal of the membrane-anchoring region increased 3-FL production by approximately 12- to 20-fold. The authors attributed this improvement to enhanced FT dimerization, activity, and thermostability. These findings demonstrate that C-terminal engineering of α-1,3-FTs requires balancing soluble expression with the structural elements needed for functional dimerization.

Collectively, these studies demonstrate that efficient FL production in *E. coli* requires coordinated optimization of FT solubility, catalytic activity, expression level, gene dosage, and structural architecture while minimizing the associated burden on host cells. These enzyme-level strategies provide a foundation for further improvements through fermentation optimization and process scale-up.

**Fermentation optimization and scale-up of *E. coli* FL production.** Following strain and enzyme engineering, fermentation optimization is required to translate improved metabolic capacity into high-titer FL production. Fed-batch fermentation is advantageous because carbon-source feeding and lactose supplementation can be adjusted over time to support biomass accumulation while maintaining optimal conditions for FL production (Table 1).

Carbon source selection and substrate feeding strategies can substantially influence FL production. Jung *et al*. compared glycerol- and glucose-based cultivation for 3-FL production and found that glycerol supported higher 3-FL production [78]. The engineered strain produced 10.7 g/L 3-FL in glycerol-based batch cultivation, whereas the corresponding glucose-utilizing strain produced 4.50 g/L. Subsequent glycerol-limited fed-batch cultivation increased the 3-FL titer to 11.5 g/L, with a yield of 0.39 mol/mol lactose and a productivity of 0.22 g/L/h. These results highlight the importance of carbon-source selection in supporting efficient carbon flux toward FL biosynthesis.

Controlled feeding of carbon sources and lactose has enabled substantially higher FL titers at larger cultivation scales. Ni *et al*. optimized glycerol feeding, induction conditions, and cultivation temperature in an engineered 2′-FL-producing strain [1]. In a 50-L fed-batch process, glycerol was continuously supplied as the main carbon source, whereas lactose was intermittently added to sustain 2′-FL production. Increasing the induction temperature from 25 to 28°C and the IPTG concentration from 0.2 to 0.3 mM increased the 2′-FL titers from 45.6 to 66.8 g/L. Under the optimized conditions, the strain achieved a yield of 0.89 mol 2′-FL/mol lactose and a productivity of approximately 0.95 g/L/h, indicating that coordinated optimization of feeding and induction conditions can support high-titer production at increased bioreactor scale.

Mengli Li *et al*. further examined substrate-feeding strategies for both 2′-FL and 3-FL production [14]. During fed-batch fermentation in 3-L bioreactors, glycerol was supplied by pH-stat feeding after induction, while lactose was intermittently supplemented to maintain its concentration between 2 and 12 g/L. Under these conditions, the optimized strains produced 64.62 g/L 2′-FL and 40.68 g/L 3-FL after 100 h, with molar yields of 0.65 mol 2′-FL/mol lactose and 0.67 mol 3-FL/mol lactose, respectively. These results indicate that coordinated control of carbon and acceptor-substrate supply is important for high-level production of both FL isomers.

Alternative carbon sources can also reduce dependence on externally supplied lactose. Parschat *et al*. developed a lactose-independent production system in which sucrose served as the sole carbon source for intracellular lactose and 2′-FL biosynthesis [11]. The final strain produced approximately 64 g/L 2′-FL after 84 h of fed-batch cultivation. This strategy demonstrates the feasibility of using sucrose as a single feedstock for both intracellular lactose formation and subsequent 2′-FL biosynthesis.

More recent studies have further increased 2′-FL production by combining extensively engineered strains with optimized fed-batch processes. Liu *et al*. achieved 79.23 g/L 2′-FL with a productivity of 1.45 g/L/h [45]. Chen *et al*. obtained 76.9 and 50.1 g/L 2′-FL from optimized plasmid-based and plasmid-free *E. coli* MG1655 strains, respectively, during fed-batch cultivation [69]. N. Li *et al*. produced 141.27 g/L 2′-FL within 45 h, corresponding to a productivity of 3.14 g/L/h [18]. Collectively, these studies illustrate the progressive improvement in 2′-FL titer and productivity achieved through the integration of strain engineering with fermentation process optimization.

Fed-batch fermentation has also enabled high-level 3-FL production, although reported titers generally remain lower than those achieved for 2′-FL. Ni *et al*. produced 35.72 g/L 3-FL in a 5-L bioreactor, with a yield of 0.82 mol/mol lactose [58]. M. Li *et al*. subsequently achieved 40.68 g/L 3-FL in a 3-L fed-batch process, with a lactose-based yield of 0.67 mol/mol [14]. Chen *et al*. developed a plasmid- and antibiotic-free production strain that generated 28.13 g/L 3-FL after 60 h in a 5-L fed-batch bioreactor, with a productivity of 0.54 g/L/h and a yield of 0.95 mol/mol lactose [77]. These studies indicate that scalable 3-FL production requires consideration of not only titer and productivity but also substrate conversion efficiency and genetic stability.

Collectively, high-titer FL production depends on both the metabolic capacity of the engineered strain and appropriate fermentation conditions. Controlled carbon-source feeding, maintenance of suitable lactose concentrations, and optimization of induction and cultivation temperature have been effective for improving FL production. Alternative feedstocks and plasmid-free production systems may further improve process robustness and economic feasibility. Nevertheless, most reported FL fermentation processes remain at laboratory scale or have been demonstrated only at limited larger scales. Further scale-up will require optimization of oxygen transfer, substrate feeding, metabolic burden, strain stability, and downstream product recovery.

### Metabolic Engineering of Yeasts for FL Production

**Precursor supply and pathway optimization in yeasts.** Building on the host-specific metabolic differences described above, yeast engineering for FL production has primarily focused on establishing and optimizing the intracellular availability of GDP-L-fucose (Fig. 2B). Unlike *E. coli*, *S. cerevisiae* and *Y. lipolytica* do not possess a complete native GDP-L-fucose biosynthetic pathway, but both provide an endogenous GDP-D-mannose pool that can be redirected toward GDP-L-fucose production. Therefore, *de novo* strategies in these yeasts commonly involve reconstruction of the downstream GDP-L-fucose module, reinforcement of precursor and cofactor supply, and balanced pathway expression in line with host physiology. However, the implementation of this strategy differs between *S. cerevisiae* and *Y. lipolytica* because of their distinct metabolic backgrounds, genetic toolkits, and cultivation properties.

In *S. cerevisiae*, *de novo* GDP-L-fucose biosynthesis was first established by converting the endogenous GDP-D-mannose pool into GDP-L-fucose through heterologous expression of *E. coli* *gmd* and *wcaG*. Mattila *et al*. demonstrated this principle by reconstructing the pathway in *S. cerevisiae*, thereby resulting in intracellular accumulation of GDP-L-fucose without exogenous GDP-D-mannose supplementation. This strategy was subsequently incorporated into FL-producing strains [79]. Liu *et al*. introduced *E. coli* *gmd* and *wcaG* together with the lactose transporter Lac12 and an α-1,2-FT, establishing *de novo* 2′-FL production in *S. cerevisiae* [20]. Lee *et al*. further enhanced 2′-FL biosynthetic pathways by increasing the expression and copy number of the heterologous biosynthetic genes and using xylose as a co-substrate to support robust GDP-L-fucose supply, resulting in 25.5 g/L 2′-FL in fed-batch fermentation [21]. Xu *et al*. targeted the upstream GDP-D-mannose pool by overexpressing *SEC53* and *PSA1*, encoding phosphomannomutase and GDP-mannose pyrophosphorylase, respectively [47]. This reinforcement increased 2′-FL production from 2.54 to 3.45 g/L in batch cultivation, and subsequent process and regulatory engineering enabled 26.63 g/L in fed-batch fermentation. More recently, Xu *et al*. introduced pheromone-based dynamic regulation of the FL biosynthetic pathway, further improving the coordination between cell growth and pathway expression and achieving 32.05 g/L 2′-FL and 20.91 g/L 3-FL in fed-batch fermentation [24].

In *Y. lipolytica*, *de novo* 2′-FL production has progressed from pathway reconstruction to host-specific carbon-flux optimization. Following the early demonstration that *Y. lipolytica* could be engineered as a 2′-FL-producing yeast cell factory, Zhang *et al*. established a more efficient *de novo* 2′-FL production platform in *Y. lipolytica* by integrating modules for lactose uptake, GDP-L-fucose biosynthesis, and fucosylation, including *LAC12*, *gmd*/*gmer*, and an α-1,2-FT. Further pathway enhancement, iterative genomic integration, and NADPH regeneration enabled the engineered strain to achieve 41.10 g/L 2′-FL in fed-batch fermentation [25]. Subsequent studies exploited the distinct substrate-utilization capacity of *Y. lipolytica*, showing that mannitol supported more efficient 2′-FL production than glucose, when mannitol transport and GDP-D-mannose synthesis were optimized [26]. More recent work further improved *de novo* 2′-FL synthesis by engineering central carbon metabolism in *Y. lipolytica*. *PFK*, which encodes phosphofructokinase and controls a major glycolytic entry point, was knocked out to redirect carbon flux from glycolysis toward GDP-D-mannose and GDP-L-fucose biosynthesis. In parallel, the pentose phosphate pathway was strengthened to increase NADPH supply for the Gmer (GDP-L-fucose synthase)-catalyzed reduction step, thereby improving both precursor and cofactor availability for 2′-FL production [27]. These studies indicate that efficient 2′-FL production in *Y. lipolytica* depends not only on reconstructing the GDP-L-fucose module but also on aligning precursor supply, redox balance, substrate utilization, and central carbon metabolism with the physiology of this non-conventional yeast.

While early *S. cerevisiae* studies mainly focused on reconstructing *de novo* GDP-L-fucose biosynthesis, Yu *et al*. demonstrated that the salvage pathway could also be used to generate GDP-L-fucose for 2′-FL production [22]. By introducing *B. fragilis* *fkp*, *H. pylori* fucT2, and *K. lactis* *LAC12*, the authors enabled the conversion of exogenous L-fucose and lactose into 2′-FL. The strain produced 503 mg/L 2′-FL in fed-batch fermentation and accumulated 0.43 mg/g DCW intracellular GDP-L-fucose. In contrast to *S. cerevisiae*, *Y. lipolytica* platforms have primarily relied on *de novo* GDP-L-fucose biosynthesis.

Lactose availability is another important constraint in yeast-based FL production, but its engineering logic differs from that in *E. coli*. Whereas *E. coli* requires balancing LacY-mediated lactose uptake with suppression of LacZ-dependent lactose hydrolysis, *S. cerevisiae* and *Y. lipolytica* generally lack efficient native lactose uptake and do not naturally degrade lactose to a substantial extent. Therefore, most yeast platforms introduce the heterologous lactose transporter Lac12 from *K. lactis* to provide intracellular lactose for FT-catalyzed FL synthesis. In this context, improving lactose availability in yeasts mainly depends on establishing and optimizing lactose transport rather than preventing lactose hydrolysis.

Overall, yeast-based FL production depends on the coordinated optimization of GDP-L-fucose supply and lactose availability. While *S. cerevisiae* has been engineered through both *de novo* and salvage pathway-based routes, reported *Y. lipolytica* platforms have primarily relied on *de novo* GDP-L-fucose biosynthesis combined with host-specific optimization of precursor supply, redox metabolism, substrate utilization, and central carbon flux. In contrast to *E. coli*, strain engineering in yeasts mainly requires the introduction and optimization of heterologous lactose transport rather than suppression of lactose hydrolysis. These differences indicate that efficient FL production in yeasts requires not only pathway reconstruction but also chassis-specific balancing of donor supply, acceptor uptake, FT activity, and cultivation conditions.

**Engineering and expression of FTs in yeasts.** In addition to precursor supply, efficient FT expression is also essential for FL production in yeast hosts. Because most FTs used for FL biosynthesis are bacterial enzymes, their functional expression in yeasts can be limited by poor folding or low solubility [20, 23, 25], insufficient catalytic activity [20, 47], and host-dependent differences in enzyme performance [23]. Accordingly, FT optimization in yeasts has focused on enzyme screening, gene dosage control, and fusion tag-mediated solubility enhancement.

In *S. cerevisiae*, early studies showed that α-1,2-FT activity can be a major bottleneck in 2′-FL production. Liu *et al*. expressed *H. pylori* *fucT2* together with the *de novo* GDP-L-fucose pathway and *LAC12*, achieving a maximum 2′-FL titer of 0.51 g/L under batch conditions [20]. To test whether α-1,2-FT activity limited production, the authors co-expressed an α-L-fucosidase from *Xanthomonas manihotis* that hydrolyzes intracellular 2′-FL into L-fucose and lactose without hydrolyzing GDP-L-fucose. In this strain, 2′-FL was not detected, whereas L-fucose accumulated to 0.41 g/L, corresponding to a theoretical 2′-FL production level of 1.23 g/L. These results suggested that insufficient α-1,2-FT activity restricted 2′-FL formation in *S. cerevisiae*.

Direct modulation of FT expression has also improved 2′-FL production. Lee *et al*. modulated the chromosomal copy number of *E. coli* *wbgL*, encoding an α-1,2-FT, in *S. cerevisiae* [21]. Under batch fermentation conditions, increasing the *wbgL* copy number from one to two increased extracellular 2′-FL production from 0.87 to 1.10 g/L and improved productivity from 0.11 to 0.14 g/L/h. These findings indicate that gene dosage-dependent tuning of FT expression can enhance 2′-FL production, although excessive expression may need to be balanced against metabolic burden and host fitness.

Enzyme screening has provided another route to improve FT performance. Xu *et al*. identified the low activity of *H. pylori* FutC as a key limitation and performed BLAST-based screening using human FucT2 and FutC as query sequences [47]. Among the selected candidates, including FutAs from *Acetobacter* sp., FutBc from *Bacillus cereus*, FutBe from *Bacteroides eggerthii*, FutBu from *Bacteroides uniformis*, and FutNc from *Neocallimastix californiae*, FutBc showed the best performance in *S. cerevisiae*. Under batch conditions, the FutBc-expressing strain produced 2.54 g/L 2′-FL with a yield of 0.89 mol/mol lactose, compared with 1.41 g/L and 0.50 mol/mol for the FutC-expressing strain. Western blot analysis further showed that FutBc expression was approximately threefold higher than FutC expression, suggesting that improved active enzyme expression contributed to enhanced 2′-FL production.

Fusion tag strategies have also been used to improve FT solubility and stability, although their effects are strongly enzyme- and host-dependent. Hollands *et al*. compared several microbial α-1,2-FTs, including FutC from *H. pylori* 26695, WcfB from *B. fragilis*, and WbgL, WbwK, and WbgN from *E. coli*, in both *S. cerevisiae* and *Y. lipolytica* [23]. In *S. cerevisiae*, FutC showed the highest 2′-FL production, and N-terminal fusion of a SUMOstar^®^ tag to FutC increased 2′-FL production by approximately 30%. However, the same tag reduced WcfB activity, indicating that fusion tag effects cannot be generalized across different FTs. A similar host- and enzyme-dependent pattern was observed in *Y. lipolytica*, where FutC also showed the highest 2′-FL productivity and SUMOstar^®^ fusion increased 2′-FL production by approximately 10-fold, whereas WcfB activity was reduced.

Further optimization in *Y. lipolytica* reinforced the importance of tag selection. Zhang *et al*. evaluated several N-terminal fusion tags, including SUMO, TrxA, DsbA, GST, ubiquitin (Ub), and MBP, to improve FutC performance. Among these, Ub fusion was the most effective, increasing 2′-FL production 2.3-fold from 0.10 to 0.23 g/L [25]. In contrast, TrxA, GST, and MBP fusions resulted in no detectable 2′-FL production, again highlighting that fusion tags can either improve or impair FT function depending on the enzyme–host combination.

Although yeast-based FT engineering has mainly focused on α-1,2-FTs for 2′-FL production, *S. cerevisiae* has also been extended to 3-FL production through α-1,3-FT expression and regulation [24]. In contrast, reported *Y. lipolytica* platforms have so far mainly focused on 2′-FL production, suggesting that further α-1,3-FT screening and host-specific optimization may be needed to broaden FL production in this host.

Overall, FT engineering in yeasts requires optimization at multiple levels, including enzyme selection, gene dosage, soluble expression, host compatibility, and linkage specificity. The available studies indicate that no single FT or fusion strategy is universally optimal across yeast hosts or FL products. Future improvements will likely require integrating enzyme screening, structure-guided engineering, and expression-level tuning to better coordinate FT activity with the supply of GDP-L-fucose and intracellular lactose availability.

**Optimization of fermentation and scale-up strategies for yeasts.** Compared with *E. coli*, scale-up of yeast-based FL production requires greater consideration of host physiology, carbon-source preference, redox metabolism, and the coordination between growth and heterologous pathway activity. Because *S. cerevisiae* and *Y. lipolytica* differ markedly in glucose regulation, substrate utilization, and cofactor balance, fermentation strategies in these hosts have not simply increased substrate feeding but have instead been used to support pathway-specific precursor supply, maintain lactose availability, and improve production-phase stability (Table 2).

Initial fermentation studies demonstrated that engineered yeasts could produce 2′-FL under controlled cultivation, although early titers were relatively low. Hollands *et al*. engineered both *S. cerevisiae* and *Y. lipolytica* as 2′-FL-producing cell factories, establishing the feasibility of using yeast hosts for FL biosynthesis [23]. Yu *et al*. further evaluated a salvage pathway-based *S. cerevisiae* strain in batch and fed-batch fermentation. The strain produced 92 mg/L 2′-FL in 48-h batch cultivation and 503 mg/L 2′-FL in 120-h fed-batch cultivation with ethanol feeding [22]. Although production remained modest, this study showed that carbon-source selection could influence the performance of yeast-based FL pathways, particularly when GDP-L-fucose was supplied through a salvage route.

Later *S. cerevisiae* studies improved FL production by linking fermentation design with the metabolic requirements of *de novo* GDP-L-fucose biosynthesis. Lee *et al*. used xylose as a co-substrate to support GDP-L-fucose formation and reduce the constraints associated with glucose repression. With lactose and xylose feeding, the engineered strain produced 25.5 g/L 2′-FL with a volumetric productivity of 0.35 g/L/h in fed-batch fermentation [21]. Xu *et al*. further combined FutBc expression, enhanced GDP-L-fucose biosynthesis, and GAL80 deletion with sucrose-based fed-batch cultivation, resulting in 26.63 g/L 2′-FL with a yield of 0.85 mol/mol lactose [47]. More recently, Xu *et al*. introduced pheromone-mediated dynamic regulation to coordinate cell growth and FL pathway expression during fermentation [24]. This strategy enabled fed-batch production of 32.05 g/L 2′-FL and 20.91 g/L 3-FL, illustrating how regulatory control can improve the transition from growth to production in *S. cerevisiae*.

In *Y. lipolytica*, fermentation optimization has increasingly exploited the host’s broad substrate-utilization capacity and distinct redox metabolism. Zhang *et al*. established a *de novo* 2′-FL production platform by integrating lactose uptake, GDP-L-fucose biosynthesis, and fucosylation modules, followed by pathway enhancement, iterative genomic integration, and NADPH regeneration [25]. The engineered strain produced 41.10 g/L 2′-FL in fed-batch fermentation, demonstrating the potential of *Y. lipolytica* as a scalable non-conventional yeast chassis. Subsequent work further showed that substrate selection is particularly important in this host. Zhang *et al*. reported that mannitol and lactose supported more efficient 2′-FL production than glucose and lactose, and optimization of mannitol transport together with GDP-D-mannose synthesis increased 2′-FL production to 27.6 g/L in fed-batch fermentation [26]. More recently, Zhang *et al*. combined central carbon metabolism engineering with mixed-carbon-source fed-batch fermentation, achieving 105.88 g/L 2′-FL with a productivity of 0.98 g/L/h in a plasmid- and antibiotic-free production system [27]. This result highlights the industrial relevance of aligning strain design, carbon-source utilization, redox balance, and genetic stability in *Y. lipolytica*.

Overall, yeast fermentation studies show that high-level FL production requires more than simply increasing bioreactor scale or substrate supply. In *S. cerevisiae*, successful strategies have focused on selecting carbon sources that support GDP-L-fucose biosynthesis, reducing glucose-related regulatory constraints, and dynamically coordinating growth with pathway expression. In *Y. lipolytica*, recent progress emphasizes the value of host-specific carbon metabolism, mannitol or mixed-carbon-source utilization, NADPH regeneration, and plasmid-free strain construction. Therefore, future scale-up of yeast-based FL production will require fermentation strategies that are tailored to each yeast chassis and integrated with pathway design, cofactor balance, lactose uptake, and FT activity.

## Conclusion

Fucosylated HMOs, particularly 2′-FL and 3-FL, have attracted increasing attention because of their biological functions, commercial relevance, and potential applications beyond infant nutrition. Although these two FL regioisomers share the same monosaccharide composition, their distinct fucosylation linkages can result in different effects on intestinal barrier regulation, immune modulation, and host–microbe interactions. Therefore, microbial production platforms should be evaluated not only by titer, yield, and productivity, but also by their ability to selectively produce specific FL structures.

Recent advances in metabolic engineering have established *E. coli*, *S. cerevisiae*, and *Y. lipolytica* as major microbial platforms for FL production. In *E. coli*, high-level FL production has been enabled by reinforcing the endogenous GDP-L-fucose pathway, introducing or optimizing salvage pathway modules, suppressing competing GDP-L-fucose and lactose degradation pathways, improving lactose uptake, engineering FTs, and optimizing fed-batch fermentation. These strategies have resulted in substantial improvements in both 2′-FL and 3-FL production, demonstrating the strong potential of *E. coli* as a scalable bacterial chassis.

Yeast-based FL production follows a different engineering logic. As *S. cerevisiae* and *Y. lipolytica* lack a complete native GDP-L-fucose biosynthetic pathway and efficient lactose uptake, these hosts require reconstruction of the GDP-L-fucose module and introduction of heterologous lactose transport. In *S. cerevisiae*, both *de novo* and salvage pathway-based strategies have been explored, and recent progress has relied on precursor-supply reinforcement, FT expression tuning, dynamic regulation, and fermentation design. In *Y. lipolytica*, recent studies have primarily focused on *de novo* 2′-FL production, with improvements driven by host-specific optimization of carbon flux, redox balance, and substrate utilization. Together, these studies show that yeast hosts provide complementary advantages for FL production, but their performance depends strongly on chassis-specific pathway balancing and process optimization.

## Future Perspectives

Although substantial progress has been made in microbial FL production, several biological and process-level challenges must be addressed to advance these platforms toward broader industrial and functional-food applications. *E. coli* remains the most advanced host for FL production owing to its well-characterized metabolism, extensive genetic toolbox, native GDP-L-fucose biosynthetic capacity, and demonstrated high-titer production of 2′-FL and 3-FL. However, lipopolysaccharide (LPS)-derived endotoxins remain a concern for food, infant nutrition, and health-related applications, highlighting the potential advantages of hosts with more favorable safety profiles.

Yeast platforms, particularly *S. cerevisiae* and *Y. lipolytica*, are promising alternatives owing to their eukaryotic physiology, industrial cultivation history, and potential for food-ingredient production. However, yeast-based FL production faces several bottlenecks that differ from those in *E. coli*. Because these yeasts lack a complete native GDP-L-fucose pathway and efficient lactose uptake, they require reconstruction of GDP-L-fucose biosynthesis and introduction of heterologous lactose transporters. In addition, most FTs used for FL biosynthesis are bacterial enzymes, and their performance in yeasts can be limited by insufficient catalytic activity, low soluble expression, and host-dependent compatibility. These limitations indicate that future yeast engineering should not rely solely on pathway reconstruction but should also integrate donor supply, acceptor uptake, FT activity, and host-specific metabolic balancing.

A major remaining challenge in yeast-based FL production is inefficient FL secretion. Although a limited number of studies have reported substantial intracellular FL accumulation in engineered *E. coli* [17, 80], extracellular accumulation is the predominant distribution pattern observed in most *E. coli* production systems. Across both defined and complex cultivation media, the majority of produced FL accumulates in the culture medium [14, 51, 66, 74]. In contrast, yeast-based production systems generally exhibit lower FL secretion efficiency, resulting in greater intracellular FL retention than observed in *E. coli*. Recent studies further suggest that FL secretion in yeast is influenced by the composition of the culture medium. In engineered *S. cerevisiae*, approximately 70% of the produced FL accumulated extracellularly in complex medium, whereas 80–90%remained intracellular in minimal medium [21, 23]. Similarly, in *Y. lipolytica*, approximately 40% of the synthesized FL remained intracellular even under fed-batch cultivation, indicating that efficient FL export remains an important engineering challenge [25]. These findings indicate that extracellular FL accumulation in yeast is a multifactorial process influenced by both FL transport and cellular physiology in response to nutrient availability. Consequently, transporter engineering represents a key strategy for improving yeast-based FL production. Future studies should prioritize identifying and optimizing efficient FL exporters that maintain high secretion efficiency under minimal and industrially relevant cultivation conditions.

An alternative perspective is that intracellular FL accumulation in yeast may not necessarily represent a limitation for all applications. Instead of maximizing extracellular FL secretion, 2′-FL-containing yeast extracts could be used directly as functional feed ingredients or in prebiotic formulations. Supporting this concept, previous studies have proposed using 2′-FL-enriched yeast extracts as feed ingredients for swine and have explored their potential as feed ingredients, with techno-economic analyses highlighting the importance of production costs and product value [21, 81]. This strategy may provide a practical means of exploiting intracellularly accumulated FL while reducing the need for extensive downstream recovery and purification.

Future work should also place greater emphasis on carbon flux and redox balance engineering in yeast hosts. In *S. cerevisiae*, high-glucose conditions induce Crabtree metabolism, leading to ethanol production and reduced carbon flux toward FL biosynthesis [21]. Strategies such as glucose-limited feeding, alternative carbon sources, and dynamic pathway regulation may help balance biomass formation with FL production [21, 82, 83]. In *Y. lipolytica*, host-specific carbon metabolism, including mannitol utilization and mixed-carbon-source cultivation, has emerged as an effective strategy for enhancing precursor supply and FL production [26, 27]. Furthermore, because GDP-L-fucose biosynthesis involves NADPH-dependent reduction steps, increasing flux through the pentose phosphate pathway and other NADPH-generating pathways represents another promising approach to improve FL productivity in yeast.

In addition to further optimizing the three major hosts discussed in this review, exploring alternative microbial platforms may provide complementary opportunities for FL production. Gram-positive bacteria such as *B. subtilis* and *C. glutamicum* avoid the LPS-derived endotoxin concerns associated with *E. coli*. Engineered *B. subtilis* has achieved 88.3 g/L 2′-FL in a 3-L bioreactor without antibiotics or chemical inducers [12], whereas *C. glutamicum* has produced 21.5 g/L 2′-FL following pathway reconstruction, putative FL exporter expression, and fermentation optimization [16]. Among yeast hosts, *K. phaffii* offers another potential production platform, with recent studies demonstrating 2′-FL biosynthesis through pathway and cofactor engineering, including an orthogonal energy module based on methanol dissimilation [28-30]. Nevertheless, further evaluation of product secretion, substrate utilization, strain stability, and downstream recovery is needed to establish the industrial potential of these alternative hosts.

Across these platforms, future efforts should move beyond optimizing individual metabolic pathways toward integrated strain and process designs that improve both FL productivity and economic feasibility. Building on sucrose-based 2′-FL production [11], one promising direction is to dynamically coordinate intracellular lactose synthesis, *de novo* GDP-L-fucose biosynthesis, and FT expression to enable efficient FL production from a single, lower-cost carbon source without exogenous lactose or L-fucose. Such regulation could help balance cell growth with FL production while reducing metabolic burden. These strategies should be evaluated not only by final titer but also by product yield on substrate and volumetric productivity. Low-cost feedstocks, such as lactose-rich dairy by-products, should also be explored, although their variable composition and pretreatment requirements may affect production efficiency. Improving FL secretion could further reduce downstream processing costs by simplifying product recovery and minimizing the need for cell disruption. Importantly, recovery strategies should be tailored to the intended product specifications, as purified FLs and FL-enriched fermentation products may require different processing approaches [21, 81]. Future techno-economic analyses should compare complete production processes rather than fermentation performance alone.

Future advances in microbial FL production will depend on integrating synthetic biology, systems metabolic engineering, and bioprocess optimization in a host-specific manner. Rather than relying on a single universal production platform, different microbial hosts are expected to serve complementary roles according to their metabolic characteristics, safety profiles, and industrial applications. Such host-tailored strategies will facilitate the sustainable and economically viable production of FL and other fucosylated HMOs.

## Figures and Tables

**Fig. 1:**
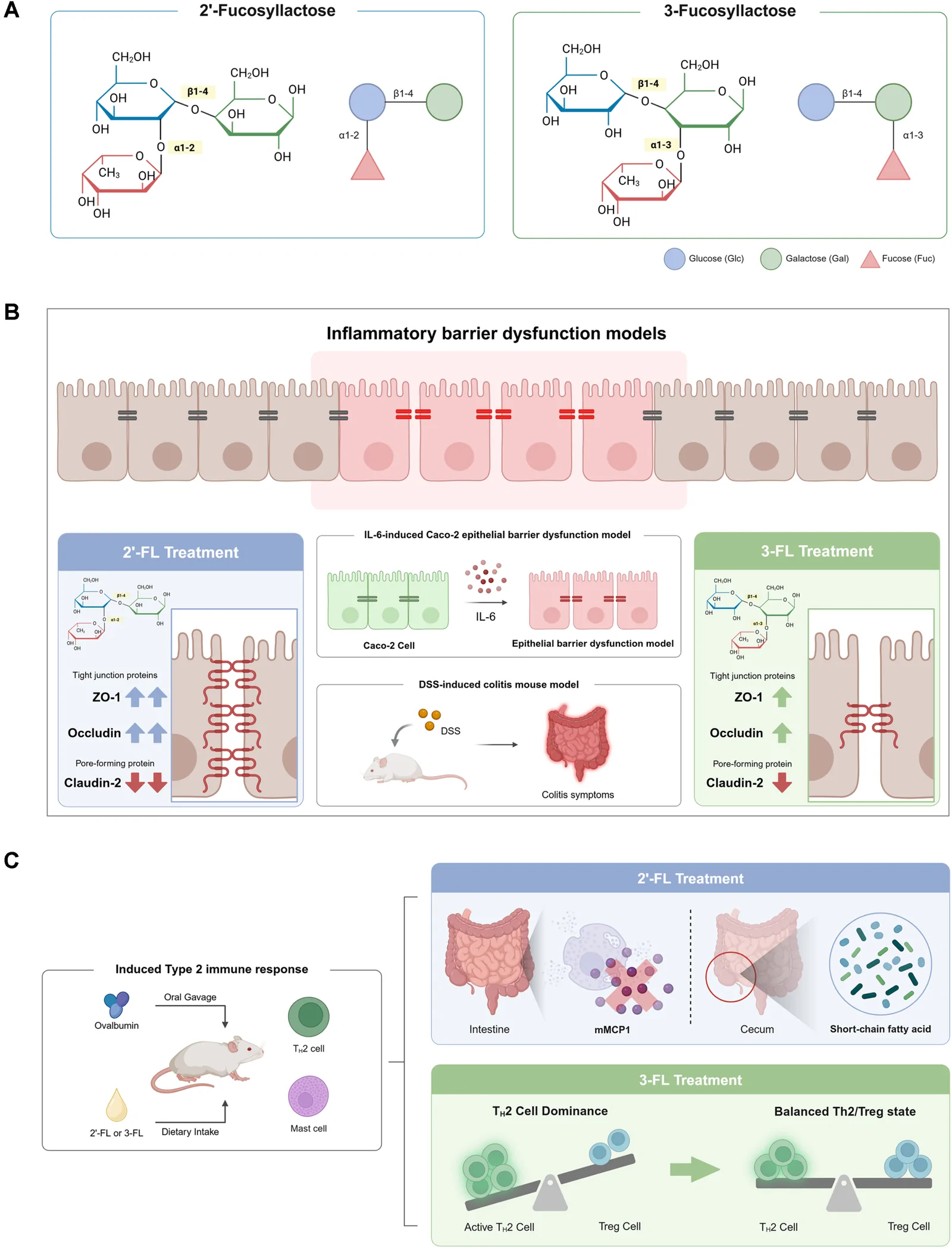
Regioisomer-specific biological functions of 2′-FL and 3-FL. (**A**) Structural comparison of 2′-FL and 3-FL. In 2′-FL, fucose is linked to the C2 position of galactose through an α-1,2 linkage, whereas in 3-FL, fucose is attached to the C3 position of the glucose moiety through an α-1,3 linkage. (**B**) Differential effects of 2′-FL and 3-FL on epithelial barrier integrity in IL-6-induced Caco-2 epithelial barrier dysfunction and DSS-induced colitis models. Both regioisomers improve barrier function, but 2′-FL shows stronger effects on tight-junction regulation, including restoration of ZO-1 and occludin and suppression of claudin-2. (**C**) Differential immunomodulatory effects of 2′-FL and 3-FL in an OVA-induced food allergy model. 3-FL modulates the Th2/Treg balance by reducing Th2 cell responses and increasing Treg cell populations, whereas 2′-FL reduces mast cell activation and increases cecal short-chain fatty acid (SCFA) levels.

**Fig. 2:**
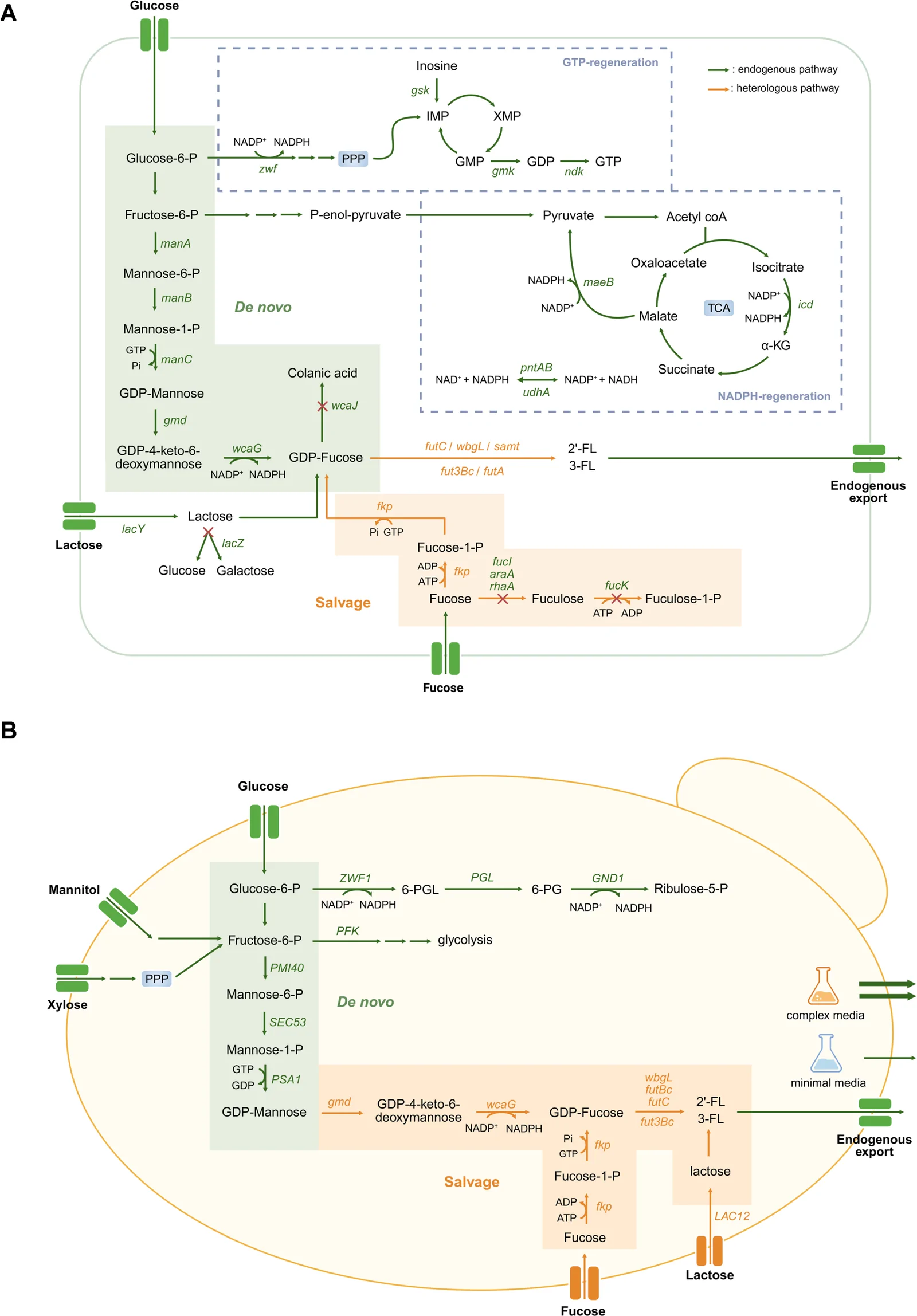
Host-specific biosynthetic pathways and metabolic engineering strategies for fucosylated HMO production. (**A**) In *E. coli*, GDP-L-fucose is synthesized through the endogenous *de novo* pathway or a heterologous salvage pathway and is transferred to lactose by α-1,2- or α-1,3-FTs to produce 2′-FL or 3-FL. Engineering strategies include reinforcement of GDP-L-fucose biosynthesis, GTP supply, and NADPH regeneration, deletion of competing pathways such as colanic acid biosynthesis and L-fucose catabolism, and optimization of LacY-mediated lactose uptake while preventing LacZ-dependent lactose hydrolysis. (**B**) In yeast hosts, the endogenous GDP-D-mannose pathway provides the precursor pool for reconstructed GDP-L-fucose biosynthesis. *S. cerevisiae* has been engineered through both *de novo* and salvage pathway-based strategies, whereas *Y. lipolytica* platforms have mainly focused on *de novo* GDP-L-fucose biosynthesis. Heterologous lactose transport, FT expression, central carbon metabolism, NADPH regeneration, and product export represent major engineering targets for improving yeast-based FL production.

**Table 1:** Representative fermentation performance of engineered *E. coli* for FL production.

Strain	Strategy	Expression	Scale	Temp.	pH	DO^a^	FL conc. (g/L)	Reference
*E. coli* C41 (DE3)	Δ*lacZ* Δ*wcaJ* Δ*nudD*; Δ*fucIK*::P_T7_-*futC*; pRS-BCGW^c^; pET-*rcsAB-futC*	Chromosomal +Episomal	50 L	37→28°C	6.8	30−50%	2′-FL: 66.80 g/L	[1]
*E. coli* BL21 (DE3)	Δ*lacZ* Δ*wcaJ* Δ*pgi*; Δ*nudD*::P_Trc_-*lacY*; Δ*lacA*::P_Trc_-*lacY*; Δ*malZ*::P_Trc_-*lacY*; pRSFDuet(P_Lac_-*setA*-P_T7_-*rcsAB*-P_T7_-*NsfutC*); pTrc99A-P_Trc_-BCGW^c^	Chromosomal +Episomal	5 L	37→30°C	6.9 ± 0.1	20−30%	2′-FL: 141.27 g/L	[18]
*E. coli* MG1655	Δ*lacZ* Δ*wcaJ*; ΔP_*lacY*_::P_J23119_; ΔP_*manC-manB*_::P_J23119_; ΔP_*gmd-fcl*_::P_J23119_; pCDFDuet-1(P_tac_-*manC-manB*; P_J23119_-*gmd-fcl*)	Chromosomal +Episomal	5 L	37→25°C	6.8 ± 0.2	~30%	2′-FL: 76.91 g/L	[69]
*E. coli* BL21 (DE3)	Δ*lacZ* Δ*wcaJ*; Δ*recA*::P_J23119_-*wbgL*; ΔP_*manC-manB*_::P_J23119_; pRSF-BCGW^c^; pET-*rcsAB-wbgL*	Chromosomal +Episomal	3 L	37→25°C	6.8	20−30%	2′-FL: 79.23 g/L	[45]
*E. coli* BL21 (DE3)	Δ*lacZ* Δ*wcaJ* Δ*nudD* Δ*pfkA* Δ*lon*; pRSF-BCGW^c^; pET-2x-(BBa_B0034)-pelB-*HpfutC*	Episomal	3 L	37→28°C	6.8	20−30%	2′-FL: 64.62 g/L	[14]
*E. coli* BL21 (DE3)	Δ*lacZ* Δ*araA* Δ*fucIK* Δ*wzxC-wcaJ* Δ*glk* Δ*pfkA* Δ*gcd* Δ*ptsG*; chr::(BCGW^c^, *wbgL, lacY, cscABKR*); chr::P_tet_-*galE-pgm-pgi*; chr:: P_tet_-*malE-galTpm1141*; chr::P_tet_-*glf*	Chromosomal	3 L	N.R.	7.0	20−30%	2′-FL: 64.0 g/L	[11]
*E. coli* BL21star (DE3)	Δ*lacZ* Δ*wcaJ*; Tn7::*lacYA*; pETDuet-1-BCGW^c^, pCOLADuet-1-*fucT*	Chromosomal +Episomal	2.5 L	37→25°C	6.78−6.82	N.R.^b^	3-FL: 11.50 g/L	[78]
*E. coli* BL21 (DE3)	Δ*lacZ* Δ*wcaJ* Δ*nudD* Δ*pfkA* Δ*lon*; pRSF-BCGW^c^; pET-2x-(BBa_B0034)-pelB-*HpM32*	Episomal	3 L	37→28°C	6.8	20−30%	3-FL: 40.68 g/L	[14]
*E. coli* C41 (DE3)	Δ*lacZ* Δ*wcaJ* Δ*nudD*; pRS-BCGW^c^, pE-*m3FT-zwf-pntAB-gsk*	Episomal	5 L	37→28°C	6.8	30−50%	3-FL: 35.72 g/L	[58]
*E. coli* BL21 (DE3)	Δ*lacZ* Δ*wcaJ*; ΔP_*manA*_::P_J23119_; ΔP_*manC-manB*_::P_J23119_; ΔP_*gmd-wcaG*_::P_J23119_; Δ*arsB*::*fut3Bc* Δ*ldhA*::*fut3Bc* Δ*poxB*::*fut3Bc* Δ*manXYZ*::*fut3Bc*	Chromosomal	5 L	37→25°C	6.6 ± 0.05	30%	3-FL: 28.13 g/L	[77]

DO^a^, dissolved oxygen; N.R.^b^, not reported; BCGW^c^, *manB-manC-gmd-wcaG.*

**Table 2:** Representative fermentation performance of engineered yeasts for FL production.

Strain	Strategy	Expression	Scale	Temp.	pH	DO^a^	FL conc. (g/L)	Reference
*S. cerevisiae* W303-1a	Δ*BAR1* Δ*GAL80* Δ*GAL1* Δ*GAL7* Δ*GAL10*; Δ*GAL1,7,10*:: P_TDH3_-*LAC12*; chr::P_VRG4_-*KMFα1*; chr::P_FUS1_-*GAL4*; chr::P_GAL1,10_-*gmd-wcaG*; chr::P_GAL1_-*futBc*	Chromosomal	5 L	30°C	5.5	20%	2′-FL: 32.05 g/L	[24]
*S. cerevisiae* W303-1a	Δ*GAL80*; chr::P_GAL1_-*LAC12*; chr::(P_GAL1_-*gmd*, P_GAL10_-*wcaG*); chr::P_GAL1_-*futBc*; chr::(P_GAL1_-*SEC53*, P_GAL10_-*PSA1*)	Chromosomal	5 L	30°C	5.5	20%	2′-FL: 26.63 g/L	[47]
*S. cerevisiae* PNY1500	Δ*HIS3* Δ*URA3*; YPRCΔ15::*LAC12*; pRS413(*gmd, gmer*); pY-SUMOstar-URA-*futC*	Chromosomal +Episomal	2 L	30°C	5.5→6.3	20%	2′-FL: 15 g/L	[23]
*S. cerevisiae* D452-2	Δ*PHO13*::*XYL123*; Δ*ALD6*::*XYL123*; chr XVI::*LAC12*; *HIS3*::*gmd–wcaG*; *URA3*::*gmd–wcaG*; chr VII::*wbgL*; chr VIII::*wbgL*; *leu2*::pRS405	Chromosomal	1 L	N.R.	5.6	N.R^b^	2′-FL: 25.5 g/L	[21]
*Y. lipolytica* PO1f	3×chr::(*GMD*_Ma_, *GMER*_Hp_, Ub–*futC*, *LAC12*); chr::(*PMI40, SEC53, PSA1*); chr::(*ZWF1, PGL, GND1*)	Chromosomal	5 L	N.R	~5.5	20%	2′-FL: 41.10 g/L	[25]
*Y. lipolytica* PO1f	Δ*PFK*; chr::(*LAC12, gmd–gmer*, SUMO-*futC*); chr::(*gmd–gmer*, SUMO-*futC, Pgl*)	Chromosomal	5 L	30°C	N.R^b^	>20%	2′-FL: 105.88 g/L	[27]
*Y. lipolytica* Y2224	Δ*URA3*; 2×chr::(*GMD*_Ma_, *GMER*_Ma_, *LAC12*, SUMOstar-*futC*)	Chromosomal	2 L	30°C	5.5→6.5	20%	2′-FL: 24 g/L	[23]
*S. cerevisiae* W303-1a	Δ*BAR1* Δ*GAL80* Δ*GAL1* Δ*GAL7* Δ*GAL10*; Δ*GAL1,7,10*:: P_TDH3_-*LAC12*; chr::P_VRG4_-*KMFα1*; chr::P_FUS1_-*GAL4*; chr::P_GAL1,10_-*gmd-wcaG*; chr::P_GAL1_-*fut3Bc*	Chromosomal	5 L	30°C	5.5	20%	3-FL: 20.91 g/L	[24]
*Y. lipolytica* PO1f	Δ*YHT3*; multicopy chr::(*LAC12, GMD, GMER, futC*), chr::*YHT1*, chr::(*SEC53, PSA1*)	Chromosomal	5 L	30°C	5.0	N.R.^b^	2′-FL: 27.6 g/L	[26]

DO^a^, dissolved oxygen; N.R.^b^, not reported.
